# Neuroprotective Effects of Exogenous Irisin in Kainic Acid-Induced Status Epilepticus

**DOI:** 10.3389/fncel.2021.738533

**Published:** 2021-10-01

**Authors:** Yao Cheng, Yaru Cui, Yujie Zhai, Wenyu Xin, Yan Yu, Jia Liang, Shucui Li, Hongliu Sun

**Affiliations:** School of Pharmaceutical Sciences, Binzhou Medical University, Yantai, China

**Keywords:** irisin, status epilepticus, mitophagy, mitochondrial oxidative stress, neuronal injury

## Abstract

Elevated reactive oxygen species (ROS) level is considered a crucial causative factor for neuronal damage in epilepsy. Irisin has been reported to ameliorate mitochondrial dysfunction and to reduce ROS levels; therefore, in this study, the effect of exogenous irisin on neuronal injury was evaluated in rats with kainic acid (KA)-induced status epilepticus (SE). Our results showed that exogenous irisin treatment significantly increased the expression of brain-derived neurotrophic factor (BDNF) and uncoupling protein 2 (UCP2), and reduced the levels of neuronal injury and mitochondrial oxidative stress. Additionally, an inhibitor of UCP2 (genipin) was administered to investigate the underlying mechanism of irisin-induced neuroprotection; in rats treated with genipin, the neuroprotective effects of irisin on KA-induced SE were found to be partially reversed. Our findings confirmed the neuroprotective effects of exogenous irisin and provide evidence that these effects may be mediated via the BDNF/UCP2 pathway, thus providing valuable insights that may aid the development of exogenous irisin treatment as a potential therapeutic strategy against neuronal injury in epilepsy.

## Graphical Abstract

Our study confirmed significant anti-oxidative stress and neuroprotective effects of exogenous irisin in KA-induced SE. Moreover, the BDNF/UCP2 pathway may contribute to the mechanism underlying the neuroprotective effects of irisin.

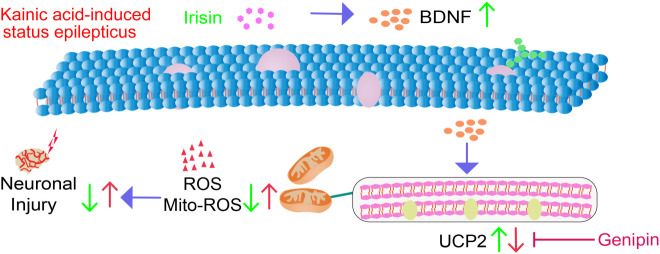

## Introduction

Epilepsy is a complex form of neuroencephalopathy that manifests clinically as disorders related to autonomous consciousness, limb tremors, and other paroxysmal movements (Balestrini and Sisodiya, 2017; Wang and Chen, 2019). As a critical pathological characteristic, neuronal injury plays a vital role in epileptogenesis, seizures, and cognitive defects (Wong, 2008), and increased levels of reactive oxygen species (ROS) are an important contributor to neuronal damage in epilepsy (Mittler, 2017). ROS are important cell-signaling molecules that are considered toxic initiators of oxidative stress (Singh et al., 2019; Tang et al., 2020). They are also closely related to mitochondrial dysfunction, and due to their high levels of energy consumption (Waldbaum and Patel, 2010b), neurons, as well as their mitochondria, are very sensitive and vulnerable to ROS (Zorov et al., 2014; Chistiakov et al., 2018). Conversely, mitochondrial dysfunction further aggravates energy deficits and induces ROS production (Waldbaum and Patel, 2010b). Consequently, ROS-induced oxidative stress is a vital contributor to neuronal injury, which in turn is critical to epileptic network formation (Waldbaum and Patel, 2010a; Dhukhwa et al., 2019).

Irisin, a novel cytokine mainly expressed in skeletal muscle and the brain (Zhang and Zhang, 2016), can provide significant protection against oxidative injury (Ren et al., 2019; Bi et al., 2020). Irisin is derived from fibronectin type-III domain-containing protein 5 (FNDC5), which is also mainly expressed in human skeletal muscle and the brain (Zhang and Zhang, 2016). In skeletal muscle after exercise, peroxisome proliferator-activated receptor-γ co-activator 1α (PGC-1α) hydrolyzes FNDC5 to irisin (Wrann et al., 2013). Certain endurance exercises have been reported to increase the expression of FNDC5 and PGC-1α, resulting in the production and release of irisin (Jedrychowski et al., 2015). Subsequently, irisin elevates the expression of brain-derived neurotrophic factor (BDNF) (Huang et al., 2019; Zarbakhsh et al., 2019; Natalicchio et al., 2020), which is widely expressed in the brain and plays a key role in the development and functioning of the nervous system (Miranda et al., 2019). BDNF can promote neuronal survival, migration, dendritization, synaptic formation, and plasticity (Bjorkholm and Monteggia, 2016). Studies have confirmed that neuronal death in the hippocampus is significantly attenuated in irisin-treated ischemic mice (Yu et al., 2020). Additionally, FNDC5, the precursor of irisin, was also confirmed to upregulate the level of BDNF (Yu et al., 2020). These findings suggest a possible neuroprotective effect of the FNDC5/irisin/BDNF pathway.

Uncoupling protein 2 (UCP2) is one of the downstream proteins whose expression is regulated by BDNF (Sepehr et al., 2020). UCP2 is a mitochondrial inner membrane protein that is mainly expressed in the brain (Toda and Diano, 2014) and is known to have neuroprotective effects (Normoyle et al., 2015). In acute seizure models, the increased expression of UCP2 has been shown to lead to a significant promotion in the survival of mitochondria and neurons (Simeone et al., 2014). As a mitochondrial negative ion carrier protein, UCP2 sends out stress signals when neurons are damaged (Barnstable et al., 2016), dissipates the ion gradient of the mitochondrial membrane, and then uncouples oxidative phosphorylation from ATP production (Toda and Diano, 2014; Dutra et al., 2018). According to recent studies, proton leakage is inextricably linked to ROS production in the mitochondrial respiratory chain (Cadenas, 2018). Reduction of mitochondrial membrane potential is not conducive to energy dissipation and stimulates the formation of ROS (Song et al., 2017). It has also been found that UCP2 can stimulate ATP production from ADP and promote proton leakage (Toda and Diano, 2014; Cadenas, 2018); the mitochondrial membrane potential increases, eventually reducing ROS generation. These results suggest the possibility of UCP2 having a potentially protective effect by decreasing ROS levels (Cadenas, 2018). Indeed, it has been reported that the increased expression of UCP2, which is induced by BDNF, regulates mitochondrial energy metabolism to reduce ROS levels (Aslan et al., 2015) and promotes neuronal survival (Varela et al., 2016).

Based on these observations from previous studies, we hypothesized that irisin may exert neuroprotective effects by attenuating oxidative stress-induced injury through the BDNF/UCP2 pathway in kainic acid (KA)-induced status epilepticus (SE). Consequently, we evaluated the role and possible mechanism of action of irisin against neuronal injury in KA-induced SE.

## Materials and Methods

### Animals and Surgical Procedures

Sprague-Dawley male adult rats (age: 8 weeks; weight: 300–310 g; Pengyue Experimental Animal Center, No. SCXK 2017-0002, Jinan, China) were used in our experiments. The animals were grouped randomly and fed with water and food *ad libitum*. All experiments were conducted in accordance with the National Institutes of Health Guidelines for the Care and Use of Laboratory Animals (National Institutes of Health Publication No. 80-23, 1996 Revision) and the Animal Ethics Regulations of the Experimental Animal Center of Binzhou Medical College (approval no. 2019002).

The animals were anesthetized using sodium pentobarbital (50 mg/kg, i.p.; CAS: 57-33-0; Xiya Reagents, China) and then fixed on a brain stereotaxic device (Anhui Zhenghua Biological Instrument Equipment Co., Ltd., China). After accurate positioning, a cannula (RSD Life Science, China) was implanted into the lateral ventricle (anteroposterior: −1.0 mm, mediolateral: −1.8 mm, dorsoventral: −3.6 mm relative to the bregma; The Rat Brain in Stereotaxic Coordinates, the third edition). The rats were allowed to recover for 5 days after surgery.

### Kainic Acid-Induced Status Epilepticus and Drug Treatments

Status epilepticus was induced using KA, a glutamate analog (Zhang et al., 2020b). A microsyringe was used to inject KA (1.25 mg/mL, 3.25 × 10^–3^ mg/kg; CAS: 58002-62-3; Sigma, United States) into the lateral ventricle through the implanted cannula to induce SE. The SE was terminated by diazepam (1 mg/mL dissolved in saline, 2 mg/kg intraperitoneally; CAS, 439-14-5, Sigma-Aldrich, United States) at 60 min after KA injection. The standard of SE was the follows: the accumulated duration of epileptic discharge by electroencephalogram (EEG) recording, accompanied with a behavioral seizure of stage 4 or stage 5 (Racine, 1972) for at least 30 min in 1 h of investigation (Trinka et al., 2015; Sharma et al., 2018; Wu et al., 2020; Zhang et al., 2020). Rats in the Untreated group were injected with saline in the same manner. Based on previous reports (Nam et al., 2010; Zhu et al., 2015; Ding et al., 2019; Ren et al., 2019; Flori et al., 2021) and our preliminary experiment, in the Irisin + KA group (*n* = 20), irisin (49.5 μg/kg; Xingbao Biotechnology Co., Ltd., China) was injected into the lateral ventricle 30 min before KA injection. Genipin (8.25 μg/kg; CAS: 6902-77-8; Aladdin, China), a specific inhibitor of UCP2, was injected into the lateral ventricle 30 min before irisin (60 min before KA) administration in the Genipin + Irisin + KA group (*n* = 20). In the corresponding control groups (Saline + KA group, *n* = 20; Saline + Irisin + KA group, *n* = 20), saline was injected instead. All drugs were dissolved with 0.9% normal saline. A total of 144 rats were used in this study; six rats in the Saline + KA group died before further analysis due to severe seizures.

### Immunohistochemistry

At 24 h and 3 days after KA administration, five rats from each group were anesthetized using pentobarbital sodium (50 mg/kg, i.p.; Xiya Reagent, China) and then perfused with 250 mL 0.9% saline, followed by 250 mL 4% paraformaldehyde. After cardiac perfusion, the brain was removed and soaked in paraformaldehyde for 24 h, then in 15% sucrose for 24 h (Zhang et al., 2020c), and subsequently in 30% sucrose until it sank to the bottom. Frozen brain tissue sections (12 μm, 50 pieces per sample) approximately posterior 2.3 mm to 5 mm relative to the bregma were prepared using a cryomicrotome (CM1850; Leica, Germany). The sections were rinsed thrice with 0.01 M phosphate-buffered saline (PBS) and subsequently incubated in 10% bovine serum albumin (MB4219-2, Dalian Meilun Biotechnology, China) for 1 h at 37°C. Primary mouse monoclonal anti-BDNF (1:200; ab205067; Abcam, United Kingdom), anti-TOMM20 (1:200; ab56783; Abcam, United Kingdom), and rabbit monoclonal anti-LC3B (1:200; ab48394; Abcam, United Kingdom) antibodies were added to the brain sections (50 μL/section, 37°C for 1 h), followed by overnight incubation at 4°C. All sections were washed with 0.01 M PBS thrice and then incubated in the dark with 50 μL fluorescein isothiocyanate-conjugated goat anti-mouse IgG (1:200; A0562; Beyotime, China) or Cy3-conjugated anti-rabbit IgG (1:200; A0516; Beyotime, China) at 37°C for 1.5 h. After three PBS washes, the sections were incubated in DAPI (4′,6-diamidino-2-phenylindole, 50 μL/section, C1005; Beyotime, China) for 15 min in the dark (Zhang et al., 2020a). Subsequently, the sections were rinsed with PBS thrice and mounted using coverslips. Finally, different brain subregions were observed and images were acquired at the same exposure intensity, using a confocal microscope (LSM 880; Zeiss, Germany). Image fluorescence intensities were analyzed using ImageJ version 1.37 (National Institutes of Health, Bethesda, United States). The average of each brain sample was taken after repeating the experiment three times, and then the data of five samples were analyzed.

### Fluoro-Jade B Staining

An FJB staining kit (AG310-30MG; EMD, Millipore, United States) was used to assess neuronal injury (Ahn et al., 2019). The brain tissue samples were incubated in 5% NaOH/80% ethanol for 5 min and then in 70% ethanol for 2 min. Subsequently, the sections were transferred into distilled water for 2 min and then into 0.06% potassium permanganate solution for 15 min. After washing with distilled water for 2 min, the sections were immersed in 0.0004% FJB dye solution for 30 min at 20°C. Then, the slides were washed in distilled water for 1 min and dried at 50°C. Finally, after treatment with xylene for 5 min, the slides were mounted using neutral resin and observed under a fluorescence microscope (Olympus, Japan).

### Oxidative Stress Assessment

A 2′,7′-dichlorodihydrofluorescein diacetate (DCFH-DA) assay kit (P0011, Beyotime Institute of Biotechnology, Shanghai, China) was used to detect the ROS level (Zhang et al., 2019) at 24 h and 3 days after KA administration. Animals were anesthetized using sodium pentobarbital (50 mg/kg, i.p.), and the cortex and hippocampus were separated quickly on ice. Then the samples (1 mg tissue added 10 μL 0.01 M PBS) were quickly cut with scissors on ice and filtered (200 mesh/inch stainless steel mesh) to prepare a single cell suspension. DCFH-DA was diluted with serum-free cell culture medium (1:1000; Beyotime, S0033, China) to a final concentration 10 μM/L. Then 500 μL DCFH-DA was added per 250 μL of single cell suspension and incubated for 40 min at 37°C in the dark. After repeated washing and centrifugation with 250 μL 0.01 M PBS, the fluorescence intensity of 200 μL samples were analyzed using a fluorescence microplate reader (Synergy H1; Thermo, United States; excitation and emission wavelength: 488 and 525 nm, respectively).

### Mitochondrial Reactive Oxygen Species Analysis Based on Mitochondrial Superoxide Levels

Samples were prepared as described for DCF analysis at 24 h and 3 days. Briefly, after anesthesia, the cortex and hippocampus tissues were quickly separated on ice, single-cell suspensions were prepared, and mitochondrial superoxide (Mito-SOX) working solution (1 mL, 5 μM; M36008, Molecular Probes, United States) was added to each 250 μL sample followed by incubation at 37°C for 10 min in the dark. The suspension was washed and centrifuged repeatedly with 0.01 M PBS, after which the fluorescence intensity was measured using a fluorescence microplate reader and flow cytometer (BD FACSCanto^TM^; BD Biosciences, United States; excitation and emission wavelength: 510 and 580 nm, respectively).

### Western Blotting

At 24 h and 3 days after KA administration, hippocampus and cortex tissues were quickly removed on ice after anesthesia. RIPA lysis buffer (MA0151, Dalian Meilun Biotechnology, China) and protease inhibitor (phenylmethanesulfonyl fluoride, ST506, Beyotime, China) were mixed at 100:1 ratio, and the tissue was added to the mixture (10 μL mixture per 1 mg brain tissue). Protein concentration was determined using a BCA Protein Assay Kit (P0012; Beyotime, China) and adjusted the same concentration. Equal amounts of protein (40 μg) were separated by 12% polyacrylamide gel electrophoresis and transferred onto a polyvinylidene fluoride membrane. After blocking for 2 h in 5% skim milk, the membranes were incubated overnight at 4°C with mouse monoclonal antibodies against BDNF (1:1000; ab205067; Abcam, United Kingdom) and Bax (ab77566; 1:1000; Abcam, United Kingdom), anti-rabbit UCP2 (1:2000; ab97931; Abcam, United Kingdom), caspase-3 (1:1000; 9662; Cell Signaling Technology, United States), activated caspase-3 (1:1000; ab2302; Abcam, United Kingdom), glyceraldehyde-3-phosphate dehydrogenase (GAPDH; 1:1000; AB-P-R 001; Kangchen, China), and Bcl-2 (ab32124; 1:1000; Abcam, United Kingdom) as appropriate. Thereafter, the membranes were incubated with horseradish peroxidase-conjugated anti-IgG secondary antibodies for 1.5 h. Finally, images were acquired and analyzed using an infrared imaging system (Odyssey, LI-COR Biosciences, United States). Protein expression in each group were compared at each timepoint, normalized to GAPDH (Sun et al., 2018). All blotting experiments were conducted under uniform conditions.

### Statistical Analyses

The sample size was estimated by balanced one-way analysis of variance (ANOVA), based on the data obtained from the preliminary experiment. All data were analyzed using SPSS (version 25.0, IBM, United States), and one-way ANOVA with Dunnett’s T3 *post hoc* test was used. All data were expressed as means ± standard errors (SEMs). For all analyses, *P* < 0.05 was considered statistically significant.

## Results

### Exogenous Irisin Treatment Increased the Expression of Brain-Derived Neurotrophic Factor and Uncoupling Protein 2 in Rats With Kainic Acid-Induced Status Epilepticus

**FIGURE 1:**
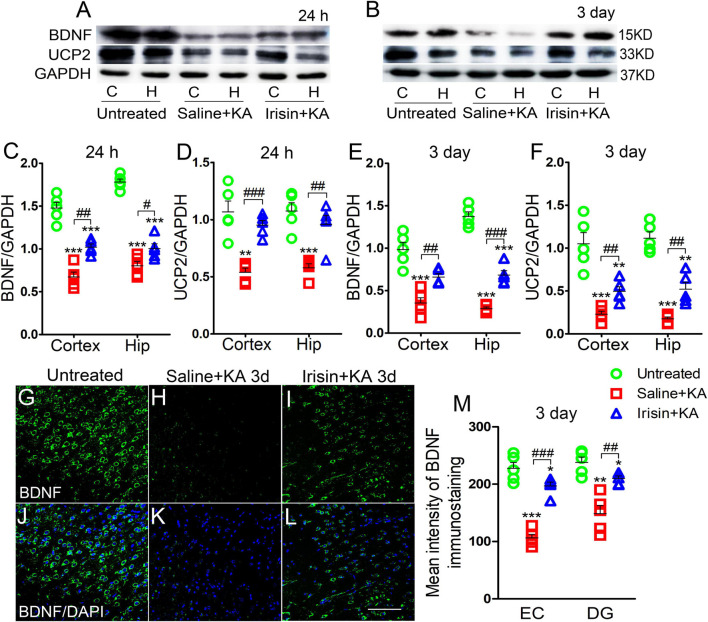
Exogenous irisin treatment increased the expression of BDNF and UCP2 in rats with KA-induced SE. **(A,B)** The expression of BDNF and UCP2 were detected by western blotting after KA treatment (*n* = 5 per group). **(C–F)** Normalized intensity of BDNF and UCP2 relative to GAPDH in the cortex and hippocampus. **(G–M)** The mean fluorescence intensity of BDNF immunostaining (green) in the left EC after exogenous irisin treatment (*n* = 5 per group). DAPI, blue. Bar = 50 μm. *P < 0.05, **P < 0.01, and ***P < 0.001, compared with controls; ^#^*P* < 0.05, ^##^*P* < 0.01, and ^###^*P* < 0.001 compared with each other (One-way ANOVA with Dunnett’s T3 *post hoc* test). C, cortex; BDNF, brain-derived neurotrophic factor; EC, entorhinal cortex; H/Hip, hippocampus; UCP2, uncoupling protein 2.

Western blotting results showed that the expression of BDNF was significantly decreased, synchronously with a reduction in UCP2 level, in the hippocampus and cortex after KA administration (*n* = 5 per group, Figures 1A–F). Immunohistochemistry results also indicated the reduced mean fluorescence intensity of BDNF in the entorhinal cortex (EC) (*n* = 5 per group, *P* < 0.001, Figures 1H,K,M), piriform cortex (PC) (data not shown), and hippocampus [e.g., dentate gyrus (DG), *P* = 0.001, Figure 1M] in rats administered KA.

Western blotting results also showed that the reduced level of BDNF in the cortex (24 h, *P* = 0.001; 3 days, *P* = 0.003; Figures 1A–C,E) and hippocampus (24 h, *P* = 0.032; 3 days, *P* < 0.001; Figures 1A–C,E) after KA administration was partly reversed in rats treated with exogenous irisin (Irisin + KA group) compared with that in rats treated with saline instead of irisin (Saline + KA group) (cortex-24 h: *P* < 0.001, 3 days: *P* < 0.001; hippocampus-24 h: *P* < 0.001, 3 days: *P* < 0.001; Figures 1A–C,E). The reduced expression of UCP2 in the hippocampus and cortex following KA administration was also partly reversed after irisin treatment (Figures 1A,B,D,F). The increased level of BDNF in the hippocampus and cortex due to exogenous irisin administration (Irisin + KA group) was also confirmed by immunohistochemistry (e.g., EC, *P* < 0.001; DG, *P* = 0.004, Figures 1G–M). These results suggest that exogenous irisin administration could increase the expression of BDNF and UCP2 in the cortex and hippocampus in KA-induced SE. However, the levels of BDNF and UCP2 in rats treated with irisin were lower than those in untreated rats (Figure 1). This indicated that the irisin treatment partly reversed the decreased levels of BDNF and UCP2 due to KA administration.

### Exogenous Irisin Treatment Alleviated Apoptosis and Neuronal Injury in Rats With Kainic Acid-Induced Status Epilepticus

**FIGURE 2:**
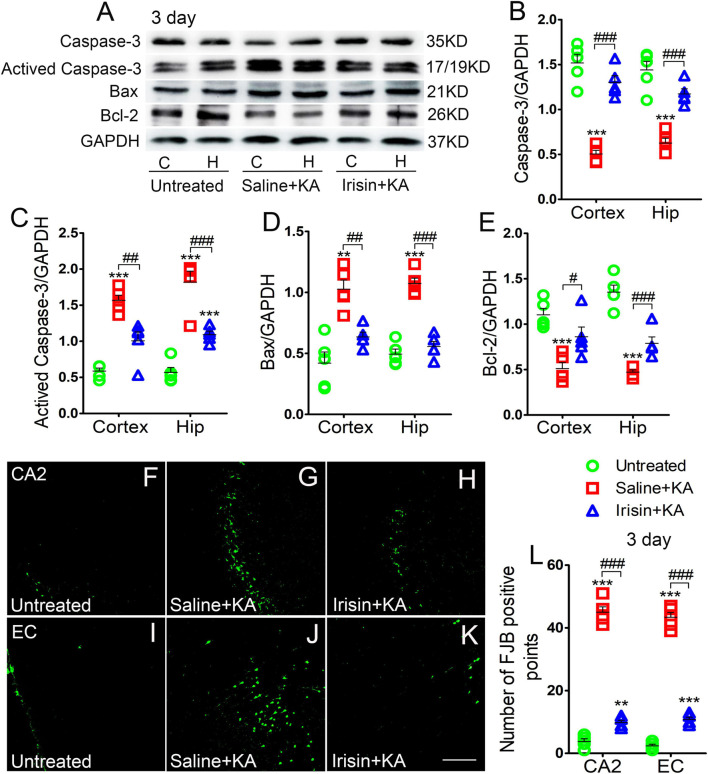
Exogenous irisin treatment alleviated apoptosis and neuronal injury in rats with KA-induced SE. **(A)** The levels of caspase-3, activated caspase-3, Bax, and Bcl-2 in the cortex and hippocampus after irisin treatment (*n* = 5/group). **(B–E)** Normalized intensity of caspase-3, activated caspase-3, Bax, and Bcl-2 relative to GAPDH. **(F–L)** Positive FJB signals in the CA2 and EC after irisin treatment (*n* = 5/group, bar = 50 μm). **P < 0.01, ***P < 0.001, compared with controls; ^#^*P* < 0.05, ^##^*P* < 0.01, and ^###^*P* < 0.001 compared with each other (One-way ANOVA with Dunnett’s T3 *post hoc* test). C, cortex; H/Hip, hippocampus; CA2, cornu ammonis 2; EC, entorhinal cortex; Bcl-2, B-cell lymphoma-2; Bax, Bcl-2-Associated X.

Expression of the positive apoptosis-related proteins, activated caspase-3 and Bax, and negative apoptosis-related caspase-3 and Bcl-2, were examined using western blotting in each group (*n* = 5 per group) at 24 h and 3 days after KA treatment. In contrast with the corresponding levels in the saline-treatment control group, the level of activated caspase-3 increased (3 day-cortex: *P* < 0.001, hippocampus: *P* < 0.001; Figures 2A,C), while that of caspase-3 decreased (3 day-cortex: *P* < 0.001, hippocampus: *P* < 0.001; Figures 2A,B). Synchronously, elevation in the level of Bax (Figures 2A,D) and reduction in that in Bcl-2 (Figures 2A,E) were detected in the cortex and hippocampus after KA administration. Similar changes of apoptosis-related proteins were found at 24 h (data not shown). However, the increased levels of activated caspase-3 and Bax induced by KA administration were alleviated by exogenous irisin treatment (3 days, Figures 2A,C,D; 24 h, data not shown). Meanwhile, irisin pre-treatment (Irisin + KA group) led to increased levels of caspase-3 and Bcl-2 compared with those in the Saline + KA group (e.g., 3 days, Figures 2A,B,E).

Neuronal degeneration was assessed based on FJB staining (*n* = 5 per group). Elevated numbers of positive signals were observed in the cortex (EC and PC) and hippocampus at 3 days after KA administration [cornu ammonis 2 (CA2): *P* < 0.001; EC: *P* < 0.001; Figures 2F–J,L]. Similar increased positive FJB signals were found at 24 h (data not shown). FJB-positive signals were significantly reduced in rats treated with irisin (Irisin + KA group) compared with those in rats treated with saline (Saline + KA group; 3 days, CA2, *P* < 0.001; EC, *P* < 0.001; Figures 2H,K,L; 24 h, data not shown). Thus, both the western blotting and FJB staining results verified that exogenous irisin treatment attenuated apoptosis and neuronal injury in rats with KA-induced SE.

### Exogenous Irisin Treatment Reduced Increased Mitophagy in Rats With Kainic Acid-Induced Status Epilepticus

**FIGURE 3:**
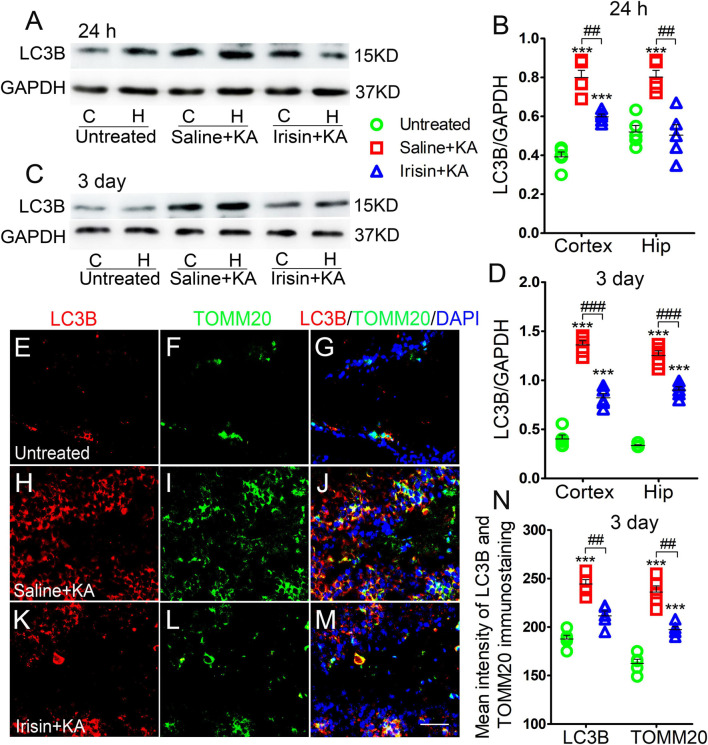
Exogenous irisin treatment reduced the increased mitophagy in rats with KA-induced SE. **(A–D)** Measurements of LC3B expression in the cortex and hippocampus after irisin administration (*n* = 5/group). **(E–N)** Mean fluorescence intensity of LC3B and TOMM20 immunostaining in the DG (*n* = 5/group). LC3B (red), TOMM20 (green), DAPI (blue). Bar = 30 μm. ***P < 0.001 compared with controls; ^##^*P* < 0.01, and ^###^*P* < 0.001 compared with each other (One-way ANOVA with Dunnett’s T3 *post hoc* test). C, cortex; H/Hip, hippocampus; LC3B, microtubule-associated protein light chain 3B; TOMM20, translocase of outer mitochondrial membrane 20; DG, dentate gyrus.

Western blotting results (*n* = 5 per group) indicated that the levels of LC3B, a marker of autophagy, increased in the cortex and hippocampus of rats treated with KA compared with those in rats treated with saline (24 h-cortex: *P* < 0.001, hippocampus: *P* < 0.001, Figures 3A,B; 3 day-cortex: *P* < 0.001, hippocampus: *P* < 0.001, Figures 3C,D). The immunohistochemistry results (*n* = 5 per group) confirmed that in rats injected with KA, the mean fluorescence intensities of LC3B and TOMM20 in the hippocampus, EC, and PC were increased. Representative results in the DG at 3 days after KA administration are presented (*P* < 0.001, Figures 3E–J,N). Moreover, the LC3B and TOMM20 positive signals overlapped almost completely (Figure 3J), suggesting that the increased autophagy in KA-induced SE is mainly mitophagy. Furthermore, western blotting results indicated that the increased level of LC3B in the hippocampus and cortex following KA administration was reduced in rats treated with irisin (Figures 3A–D). A similar reversion of LC3B and TOMM20 level by exogenous irisin treatment was also observed by immunohistochemistry (e.g., 3 days, DG, Figures 3K–N). Accordingly, our preliminary inference is that exogenous irisin can reduce mitophagic activity.

### Exogenous Irisin Treatment Attenuated the Elevated Oxidative Stress Levels in Rats With Kainic Acid-Induced Status Epilepticus

**FIGURE 4:**
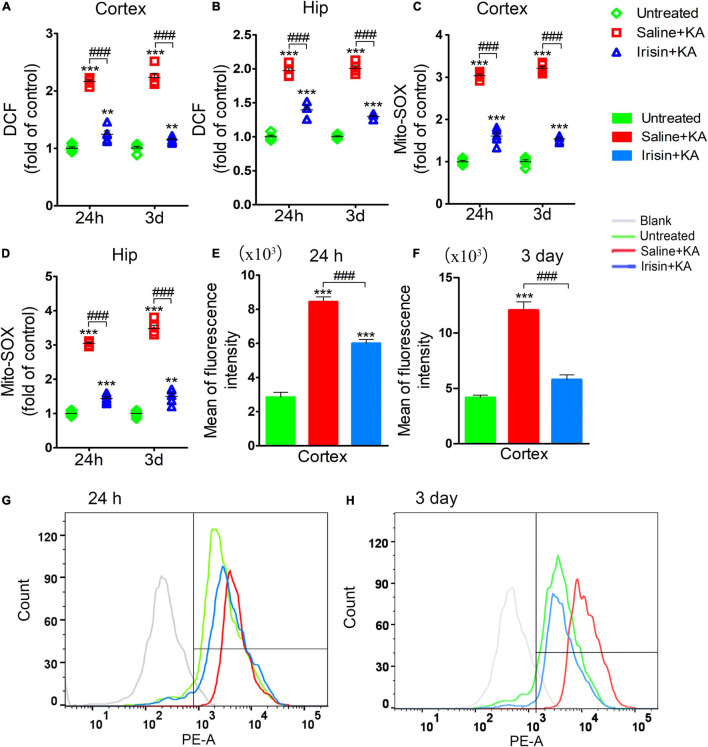
Exogenous irisin treatment attenuated the elevated oxidative stress levels in rats with KA-induced SE. The elevated levels of DCF **(A,B)** and Mito-SOX **(C,D)** in the cortex and hippocampus induced by KA administration were decreased by exogenous irisin treatment. **(E–H)** The fluorescence intensity of Mito-SOX. 24 h, *n* = 5/group; 3 days, *n* = 8/group. **P < 0.01, ***P < 0.001 compared with controls; ^###^*P* < 0.001 compared with each other (One-way ANOVA with Dunnett’s T3 *post hoc* test). Hip, hippocampus.

The degree of oxidative stress was evaluated based on DCF/Mito-SOX levels observed at 24 h (*n* = 5/group) and 3 days (*n* = 8/group) after KA administration. Increased DCF levels were detected in the cortex and hippocampus after KA administration (Figures 4A,B). Similarly, the levels of Mito-SOX also increased after KA administration (Figures 4C–H). However, irisin treatment (Irisin + KA group) led to significant reductions in the levels of DCF (Figures 4A,B) and Mito-SOX (Figures 4C,D) compared with those in rats treated with saline (Saline + KA group). Flow cytometry results showed a higher mean fluorescence intensity of Mito-SOX after KA intervention at 24 h (cortex, *P* < 0.001; Figure 4E; hippocampus, data not shown) and 3 days (cortex, *P* < 0.001; Figure 4F; hippocampus, data not shown). Conversely, the increased mean fluorescence intensity of Mito-SOX in the hippocampus and cortex at 24 h and 3 days was significantly decreased after irisin treatment compared with that in rats with KA-induced SE treated with saline (representative results in the cortex are presented in Figures 4E–H). These results indicated that exogenous irisin treatment reduced the increased oxidative stress levels induced by KA administration.

### Elevations in Uncoupling Protein 2 Levels Induced by Exogenous Irisin Administration Were Reversed by Genipin

**FIGURE 5:**
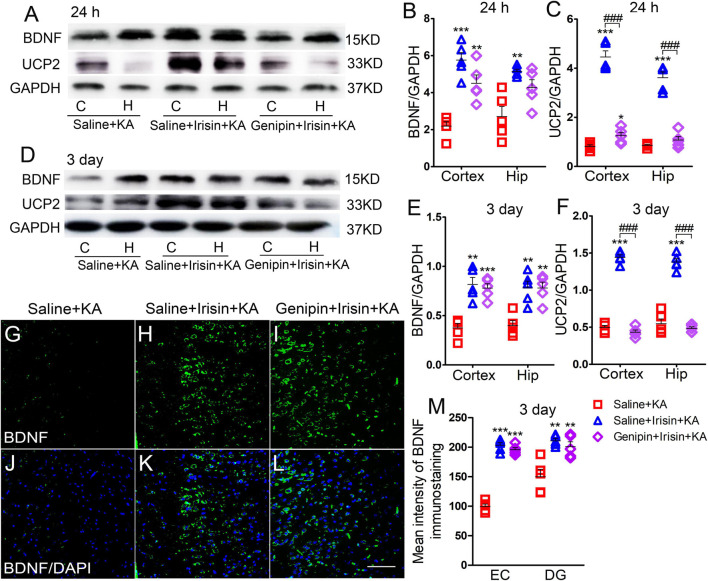
Elevations in UCP2 levels induced by exogenous irisin treatment were reversed by genipin. **(A–F)** Measurements of BDNF and UCP2 expression in the cortex and hippocampus after irisin administration (*n* = 5/group). **(G–M)** The mean fluorescence intensity of BDNF immunostaining in the left EC after genipin injection (3 days, *n* = 5/group). Bar = 50 μm. *P < 0.05, **P < 0.01, ***P < 0.001 compared with controls; ^###^*P* < 0.001 compared with each other (One-way ANOVA with Dunnett’s T3 *post hoc* test). C, cortex; BDNF, brain-derived neurotrophic factor; EC, entorhinal cortex; H/Hip, hippocampus; UCP2, uncoupling protein 2.

The expression of BDNF and UCP2 in the cortex and hippocampus at 24 h and 3 days was assessed using western blotting (*n* = 5 per group, Figures 5A–F). The expression of BDNF increased at 24 h (cortex, *P* < 0.001; hippocampus, *P* = 0.002; Figures 5A,B) and 3 days (cortex, *P* = 0.001; hippocampus, *P* = 0.001; Figures 5D,E) after exogenous irisin treatment; elevated expression of UCP2 was also observed at 24 h (Figures 5A,C) and 3 days (Figures 5D,F). However, after genipin administration (Genipin + Irisin + KA group), the expression of BDNF showed no significant variation compared with that in the Irisin + KA group (24 h, Figures 5A,B; 3 days, Figures 5D,E), but the level of UCP2 was decreased (24 h-cortex: *P* < 0.001, hippocampus: *P* < 0.001; 3 day-cortex: *P* < 0.001, hippocampus: *P* < 0.001; Figures 5A,C,D,F). Meanwhile, immunohistochemistry results also showed that no significant changes of fluorescence intensity of BDNF immunostaining in brain were observed after genipin treatment (*n* = 5 per group, e.g., EC, DG, Figures 5G–M, other regions results not shown). These results showed that the UCP2 inhibitor genipin, which can reduce the expression of UCP2, can partly reverse the protective effects of exogenous irisin.

### Genipin Reversed the Effects of Irisin on Kainic Acid-Induced Apoptosis and Neuronal Degeneration

**FIGURE 6:**
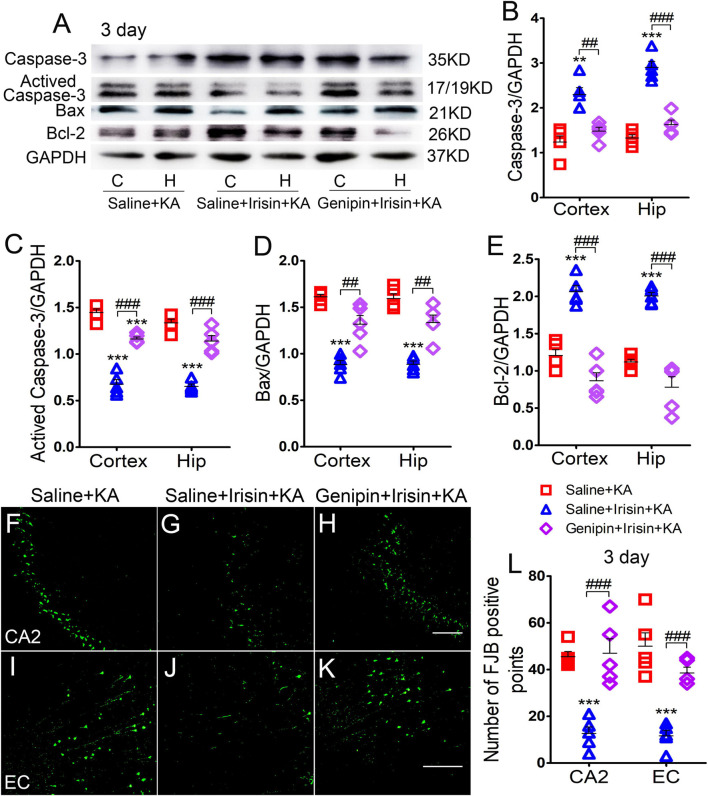
Genipin reversed the effects of irisin on KA-induced apoptosis and neuronal degeneration. **(A–E)** The immunoreactivity of caspase-3, activated caspase-3, Bax, and Bcl-2 (*n* = 5/group). **(F–L)** Positive signals of FJB in CA2 and EC (*n* = 5/group). Bar = 50 μm. **P < 0.01, ***P < 0.001 compared with controls; ^##^*P* < 0.01, ^###^*P* < 0.001 compared with each other (One-way ANOVA with Dunnett’s T3 *post hoc* test). C, cortex; EC, entorhinal cortex; H/Hip, hippocampus; CA2, cornu ammonis 2; Bcl-2, B-cell lymphoma-2; Bax, Bcl-2-Associated X.

The levels of apoptosis-related proteins were assessed using western blotting (*n* = 5 per group). At 3 days after irisin administration, the levels of caspase-3 (Figures 6A,B) and Bcl-2 (Figures 6A,E) increased, accompanied by reduced levels of activated caspase-3 (Figures 6A,C) and Bax (Figures 6A,D) in the cortex and hippocampus. After genipin treatment (Genipin + Irisin + KA group), the levels of activated caspase-3 increased (cortex: *P* < 0.001, hippocampus: *P* < 0.001; Figures 6A,C), whereas those of caspase-3 significantly decreased in the cortex (*P* = 0.001) and hippocampus (*P* < 0.001; Figures 6A,B). Synchronously, elevated levels of Bax (*P* = 0.003) and hippocampus (*P* = 0.001) and reduced levels of Bcl-2 (cortex: *P* < 0.001, hippocampus: *P* < 0.001; Figures 6A,E) were detected after genipin administration. Similar changes in apoptosis-related proteins were found at the 24 h timepoint (data not shown).

The results of FJB staining showed that the decreased FJB-positive signals due to exogenous irisin treatment (CA2: *P* < 0.001, Figures 6F,G,L; EC: *P* < 0.001, Figures 6I,J,L) increased after genipin administration (*n* = 5 per group; CA2: *P* < 0.001, Figures 6H,L; EC: *P* < 0.001, Figures 6K,L) at 3 days after KA administration. Similar changes were found at the 24 h timepoint (data not shown). The FJB staining results showed that genipin could reverse the inhibition of neuronal injury mediated by irisin. Our results indicate that UCP2 may contribute to the neuroprotective effects of irisin in KA-induced SE.

### The Reduction of Mitophagy Due to Exogenous Irisin Treatment Was Reversed After Genipin Administration

**FIGURE 7:**
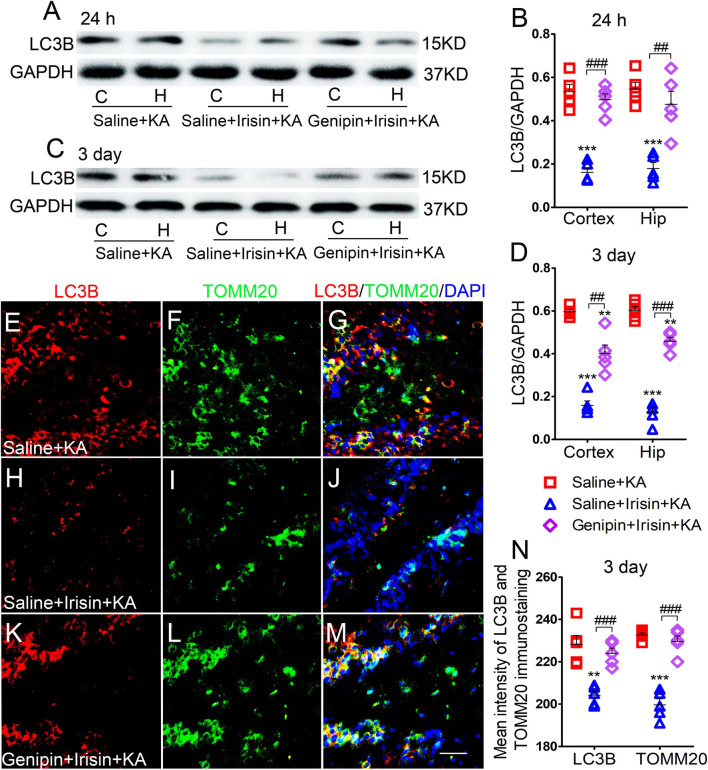
The reduction of mitophagy due to exogenous irisin treatment was reversed after genipin administration. **(A–D)** The levels of mitophagy-related protein LC3B after genipin treatment was detected by western blot (**A–D**, *n* = 5/group). **(E–N)** The mean fluorescence intensity of LC3B and TOMM20 immunostaining in DG (*n* = 5/group). LC3B (red), TOMM20 (green), DAPI (blue). Bar = 30 μm. **P < 0.01, ***P < 0.001 compared with controls; ^##^*P* < 0.01, ^###^*P* < 0.001 compared with each other (One-way ANOVA with Dunnett’s T3 *post hoc* test). C, cortex; EC, entorhinal cortex; H/Hip, hippocampus; LC3B, microtubule-associated protein light chain 3B; TOMM20, translocase of outer mitochondrial membrane 20; DG, dentate gyrus.

Western blotting analysis (*n* = 5 per group) showed that after irisin treatment, LC3B expression decreased in the cortex (24 h, *P* < 0.001; 3 days, *P* < 0.001; Figures 7A–D) and hippocampus (24 h, *P* < 0.001; 3 days, *P* < 0.001; Figures 7A–D). Immunohistochemistry analysis confirmed that the levels of LC3B and TOMM20 decreased in the hippocampus, EC, and PC at 24 h and 3 days after KA administration due to exogenous irisin treatment (3 days, DG, Figures 7E–J,N; other data not shown). However, after genipin administration, the decreased level of LC3B was reversed (Figures 7A–D). Similar changes were confirmed by immunohistochemistry (*n* = 5 per group; 3 days, DG, *P* < 0.001, Figures 7K–N; other data not shown). Thus, the reduction in mitophagy due to irisin treatment was reversed by genipin administration.

### Genipin Reversed the Reduced Level of DCF/Mito-SOX Mediated by Exogenous Irisin

**FIGURE 8:**
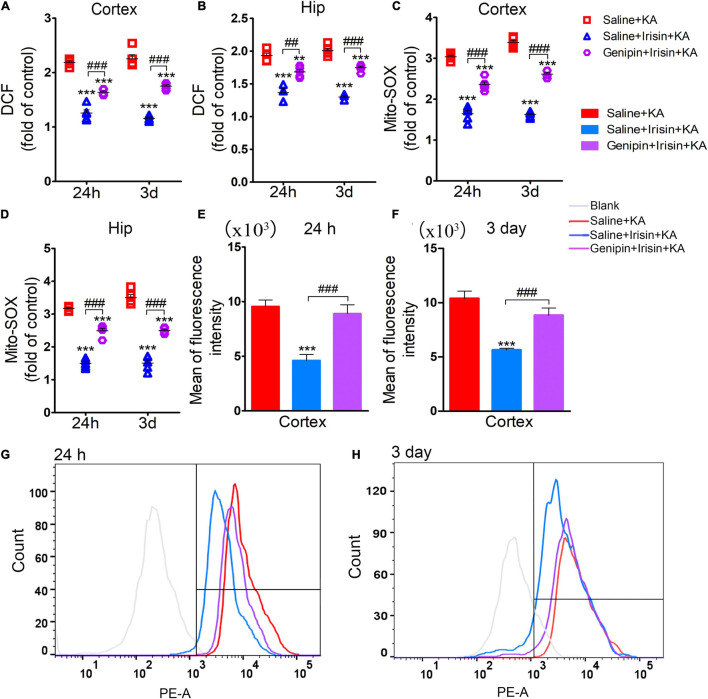
Genipin reversed the reduced level of DCF/Mito-SOX mediated by exogenous irisin. The levels of DCF **(A,B)**, Mito-SOX **(C,D)** was detected in cortex and hippocampus after genipin administration. **(E–H)** Mito-SOX levels due to genipin administration were observed based on the flow cytometry analysis. 24 h, *n* = 5/group; 3 days, *n* = 8/group. **P < 0.01, ***P < 0.001 compared with controls; ^##^*P* < 0.01, ^###^*P* < 0.001 compared with each other (One-way ANOVA with Dunnett’s T3 *post hoc* test). Hip, hippocampus.

The analysis of oxidative stress levels (24 h, *n* = 5/group; 3 days, *n* = 8/group) showed that the reduced DCF levels due to irisin treatment (Saline + Irisin + KA group) increased after genipin administration (Genipin + Irisin + KA group; 24 h-cortex: *P* < 0.001, hippocampus: *P* = 0.001; 3 day-cortex: *P* < 0.001, hippocampus: *P* < 0.001; Figures 8A,B). Meanwhile, genipin also reversed the decreased levels of Mito-SOX (24 h-cortex: *P* < 0.001, hippocampus: *P* < 0.001; 3 day-cortex: *P* < 0.001, hippocampus: *P* < 0.001; Figures 8C,D). Similar changes in Mito-SOX levels due to genipin administration were observed based on flow cytometry analysis (Figures 8E–H). These results indicated that the anti-oxidative effects of exogenous irisin were reversed by genipin administration.

## Discussion

Our study found significant neuroprotective effects exerted by exogenous irisin, as demonstrated by increased expression of BDNF and UCP2 and decreased levels of DCF/Mito-SOX, in rats with KA-induced SE. Moreover, after treatment with genipin, an inhibitor of UCP2, the neuroprotection provided by irisin was partly reduced.

Irisin, a recently discovered hormone, comprises 112 amino acids and is derived from the extracellular domain of FNDC5 (Nie and Liu, 2017). Initially, it was found that skeletal muscles release a type of hormone-like molecule that promotes white fat browning after extensive exercise (Jedrychowski et al., 2015; Colaianni et al., 2017); subsequent studies confirmed that this molecule is irisin (Zhang and Zhang, 2016), which is expressed in skeletal muscle, heart, brain, and other tissues, and is involved in macromolecular metabolism, certain mitochondrial functions (Colaianni et al., 2017), as well as glucose metabolism, immune regulation, and metabolic diseases (Polyzos et al., 2018; Yang and Leung, 2020). Mitochondrial function is very important to neuronal survival in epilepsy (Folbergrova and Kunz, 2012; Wu et al., 2019). A previous study found that mitochondrial defects, such as mitochondrial swelling, cristae lysis, and apoptosis, could be observed by electron microscopy (Finsterer and Scorza, 2017). Recent studies have shown that irisin can improve mitochondrial function and plays protective roles in various diseases by reducing ROS production (Zhang et al., 2021). Consistent with previous studies (Bi et al., 2020; Yang and Leung, 2020; Zhang et al., 2021), we confirmed that in rats with KA-induced SE, exogenous irisin treatment significantly reduced ROS and Mito-ROS levels, while neuronal injury in the hippocampus, piriform cortex, and EC was synchronously attenuated. Interestingly, the Elhady et al. (2018) found a significant elevated level of serum irisin in children with epilepsy, especially those without controlled seizures. This study suggested the contribution of irisin in epilepsy. Combined with the neuroprotective effects of irisin verified by previous studies (Islam et al., 2017) and our results, it is likely that endogenous irisin may also protect against the neuronal injury induced by epilepsy.

Irisin-mediated neuronal protection is closely related to BDNF (de Oliveira Bristot et al., 2019; Sepehr et al., 2020), which is a vital growth factor that is widely expressed in the central nervous system and plays an important role in neuronal maturation, synapse formation, and plasticity (Zaletel et al., 2017; de Oliveira Bristot et al., 2019; Sepehr et al., 2020). It has been confirmed that irisin exerts its neuroprotective effects by increasing the expression of BDNF (Huang et al., 2019). Indeed, the increased levels of both FNDC5 (the precursor of irisin) and irisin after exercise lead to elevated expression of BDNF (Gomez-Pinilla et al., 2008; Asadi et al., 2018). Our results also confirmed that the reduced expression of BDNF after KA administration was ameliorated by exogenous irisin treatment. Moreover, KA-induced neuronal damage was also attenuated in irisin-treated rats. These results suggest a possible role of BDNF in the neuroprotective effects of irisin in KA-induced SE rats.

Interestingly, the decrease in expression of BDNF after KA administration was accompanied by acute oxidative stress and neuronal injury, although the excitation of skeletal muscle during SE may result in irisin production. Our results indicate that irisin, which is expected to be derived from skeletal muscle excitation during SE, is not sufficient to protect neurons from the damage caused by overexcitation in KA-induced SE. Further studies are needed to evaluate the contribution of the irisin produced by skeletal muscles during SE.

Uncoupling protein 2 is a downstream molecule of BDNF and is closely linked to the neuroprotective effects of BDNF (Barnstable et al., 2016). UCP2 is regarded as an important signaling molecule related to neuronal survival (Dutra et al., 2018; de Oliveira Bristot et al., 2019). As an anion carrier protein located in the mitochondrial inner membrane, UCP2 is sensitive to mitochondrial membrane potential (de Oliveira Bristot et al., 2019; Sepehr et al., 2020). It has been verified that when mitochondrial membrane potential decreases, large amounts of calcium flow into the mitochondria, leading to ROS production and cell injury or even death (Nemani et al., 2018; Liu et al., 2019). Recent studies have confirmed that UCP2 reduces ROS levels by reducing the proton gradient of mitochondria, thereby reducing cell death (Cadenas, 2018). UCP2 also plays an important protective role in acute physiological stress (Cadenas, 2018) and promotes neuronal stress tolerance in certain environments (Azzu and Brand, 2010). Additionally, it has been proven that increased expression of UCP2 can improve the antioxidant capacity of aging retinal cells (He et al., 2019). In some mouse and rat models of cerebral ischemia, overexpressed UCP2 plays a neuroprotective role by regulating the expression of Bcl-2 family proteins and cell survival factors (Haines and Li, 2012). The Hirose group reported that lifespan was reduced in UCP2 deficient mice, suggesting that reduced UCP2 level may be a participative factor in the aging process (Lourenco et al., 2019). These studies suggest a possible contribution of the BDNF/UCP2 pathway in the neuroprotective effects of irisin. Consistent with previous studies, our results showed that the increased levels of ROS/Mito-SOX and neuronal injury in rats with KA-induced SE were significantly attenuated after irisin treatment, accompanied by elevated BDNF and UCP2 expression. Moreover, upon treatment with genipin, an inhibitor of UCP2, the protective effects of irisin were partly reversed—for example, the reduced oxidative stress level increased, and attenuated neuronal injury was aggravated. This suggests that irisin may prevent oxidative stress-induced neuronal injury through the BDNF/UCP2 pathway in KA-induced SE.

As a versatile regulator, Ca^2+^ is closely related to ATP synthesis, ROS production, and other homeostasis-related activities in mitochondria (Normoyle et al., 2015) which play vital roles in epilepsy (Folbergrova and Kunz, 2012; Burnstock, 2020; Kapron et al., 2020). Studies have found that elevated intracellular calcium levels in neurons can lead to seizures (Traub and Llinás, 1979; Jones, 2002), which may be due to the Ca^2+^ influx leading to activation of glutamate-associated receptors and neuroexcitatory toxicity (Mattiasson and Sullivan, 2006; Abramov and Duchen, 2008; Armada-Moreira et al., 2020). UCP2 has been reported to affect mitochondrial membrane potential by facilitating proton leakage (Mattiasson and Sullivan, 2006) and regulating the influx of Ca^2+^ associated with mitochondrial membrane (Motloch et al., 2016; Larbig et al., 2017). Combined with the possible contribution of the BDNF/UCP2 pathway in the neuroprotective effect of irisin, we speculate that mitochondrial calcium homeostasis may be related to the neuroprotective effect of irisin, which is the focus of future research.

In summary, our study confirmed significant anti-oxidative stress and neuroprotective effects of exogenous irisin in KA-induced SE. Our results also indicate that the BDNF/UCP2 pathway may contribute to the mechanism underlying the neuroprotective effects of irisin. Irisin treatment could emerge as a potential strategy against oxidative stress injury in epilepsy. Considering the close relationship between ROS levels and epileptic seizures, further studies in this regard are needed to validate and clarify the anti-seizure effects of irisin in detail.

## Data Availability Statement

The original contributions presented in the study are included in the article/Supplementary Material, further inquiries can be directed to the corresponding author/s.

## Ethics Statement

The animal study was reviewed and approved by the Animal Ethics Regulations of the Experimental Animal Center of Binzhou Medical College (approval no. 2019002).

## Author Contributions

YCh and YCu: study design, data acquisition, and manuscript drafting. YZ, WX, YY, HS, and JL: KA-induced SE model preparation, data acquisition, and analysis. SL and HS: study conception, design, and data interpretation. All authors approved the final manuscript.

## Conflict of Interest

The authors declare that the research was conducted in the absence of any commercial or financial relationships that could be construed as a potential conflict of interest.

## Publisher’s Note

All claims expressed in this article are solely those of the authors and do not necessarily represent those of their affiliated organizations, or those of the publisher, the editors and the reviewers. Any product that may be evaluated in this article, or claim that may be made by its manufacturer, is not guaranteed or endorsed by the publisher.

## Supplementary Material

Supplementary Figure 1 The original gel bands of Figure 1.

Supplementary Figure 2 The original gel bands of Figure 2.

Supplementary Figure 3 The original gel bands of Figure 3.

Supplementary Figure 4 The original gel bands of Figure 5.

Supplementary Figure 5 The original gel bands of Figure 6.

Supplementary Figure 6 The original gel bands of Figure 7.

Supplementary Figure 7 Experimental grouping and detailed pharmacological manipulations.
