# Supplementary figures and images for: Neuroprotective Effects of Exogenous Irisin in Kainic Acid-Induced Status Epilepticus
Source: Front Cell Neurosci. 2021 Oct 1;15:738533. doi: 10.3389/fncel.2021.738533 (PMC8517324; doi:10.3389/fncel.2021.738533)

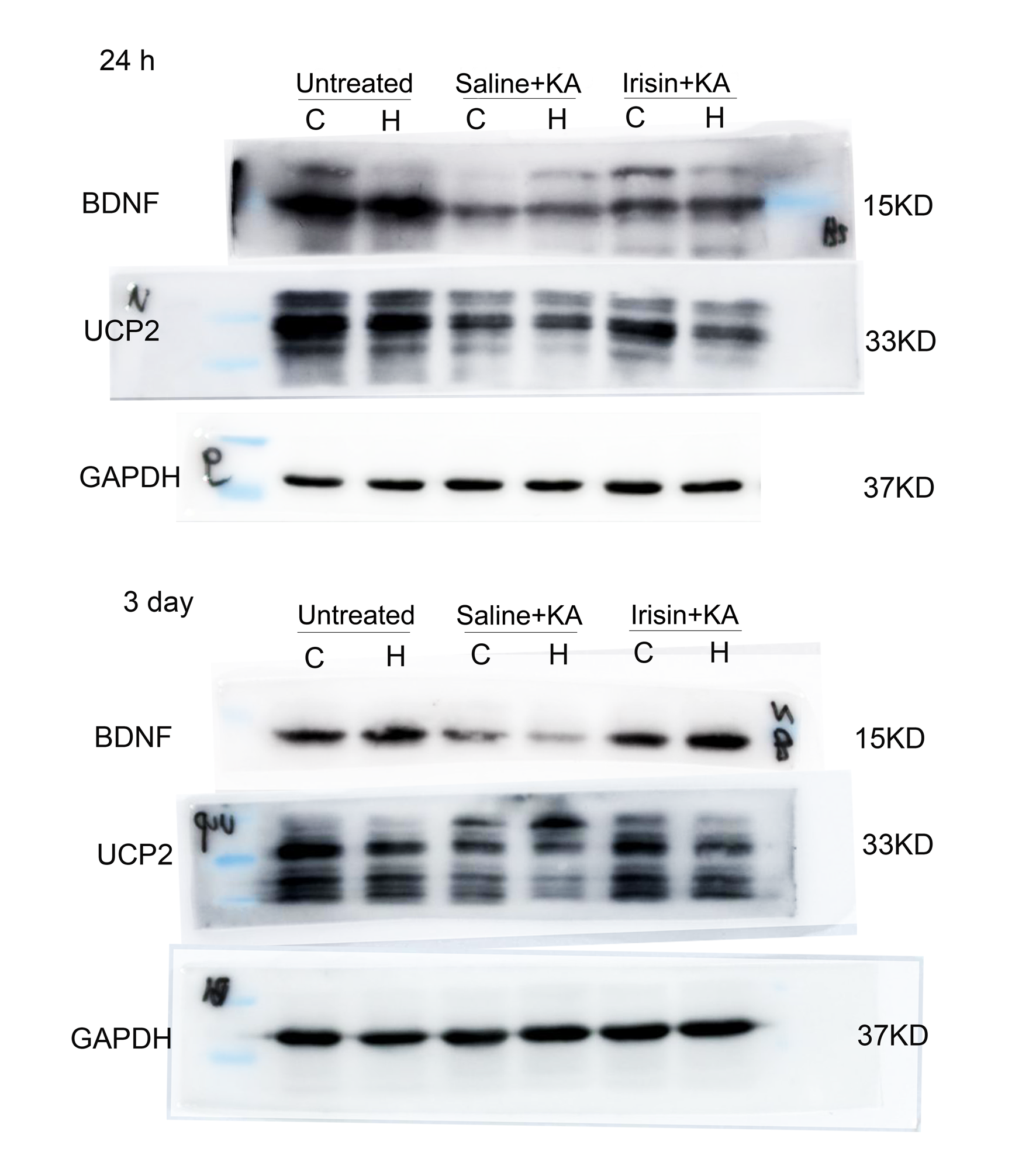

Supplement: Supplementary Figure 1 — The original gel bands of Figure 1. [file Image_1.tif]

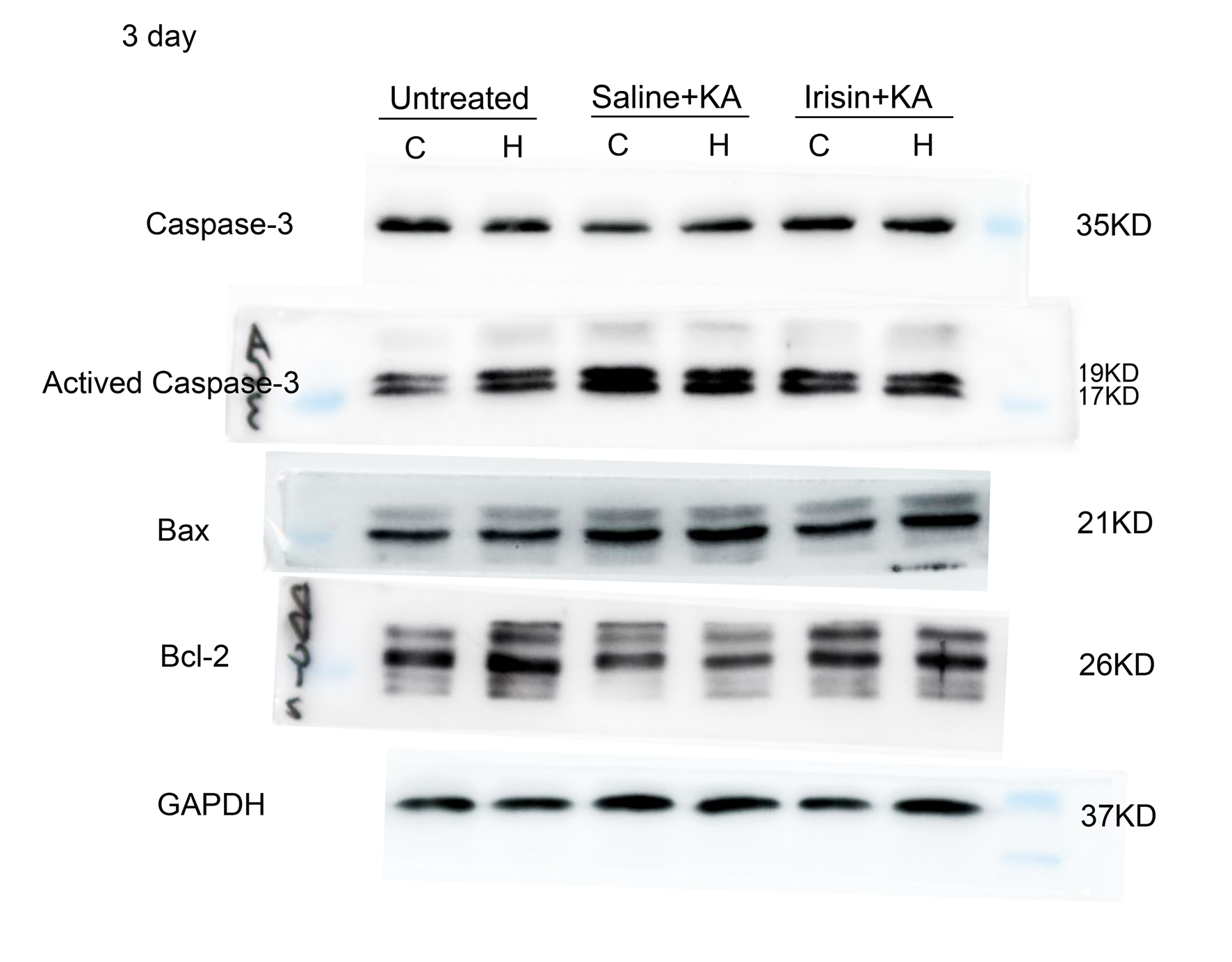

Supplement: Supplementary Figure 2 — The original gel bands of Figure 2. [file Image_2.tif]

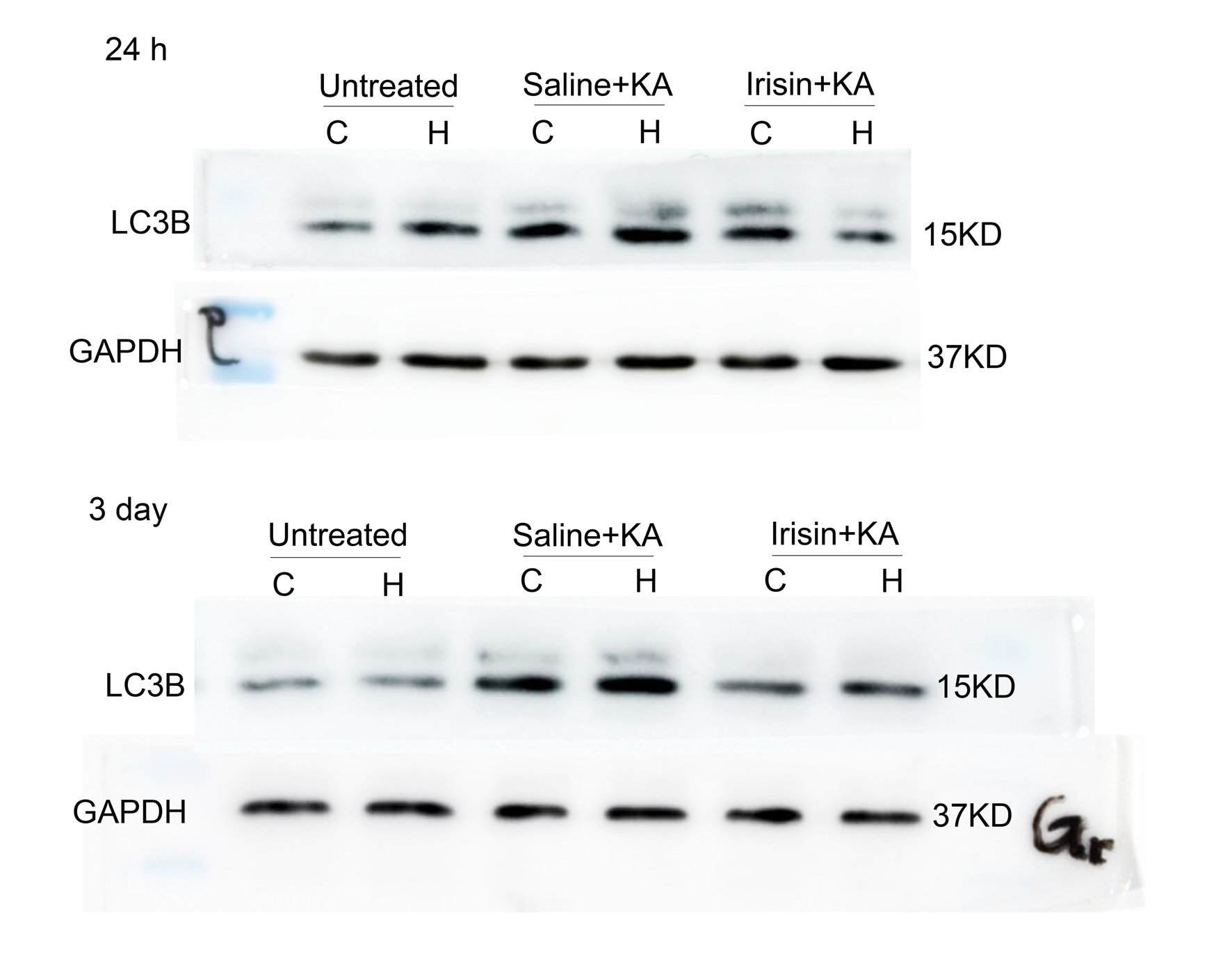

Supplement: Supplementary Figure 3 — The original gel bands of Figure 3. [file Image_3.tif]

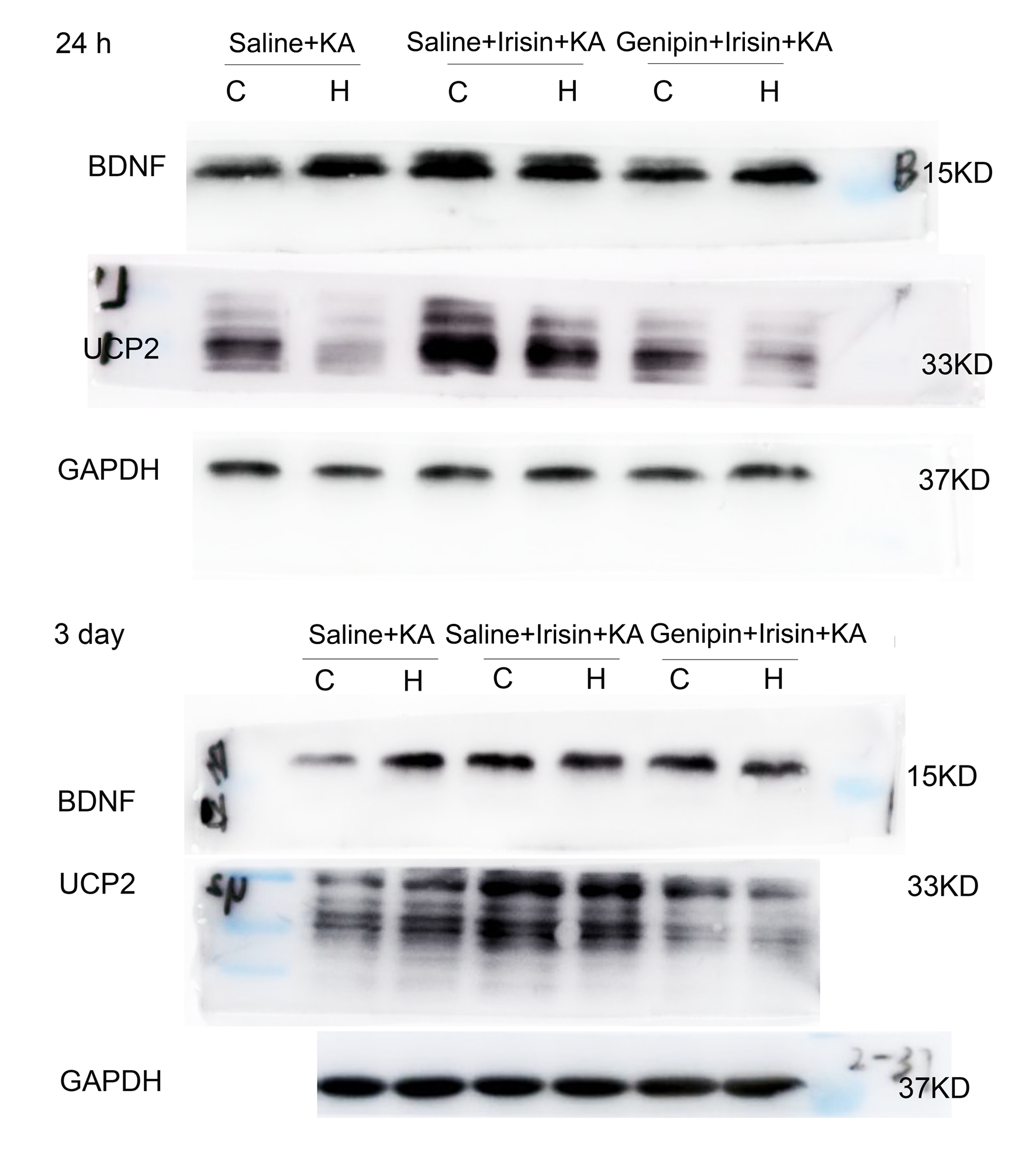

Supplement: Supplementary Figure 4 — The original gel bands of Figure 5. [file Image_4.tif]

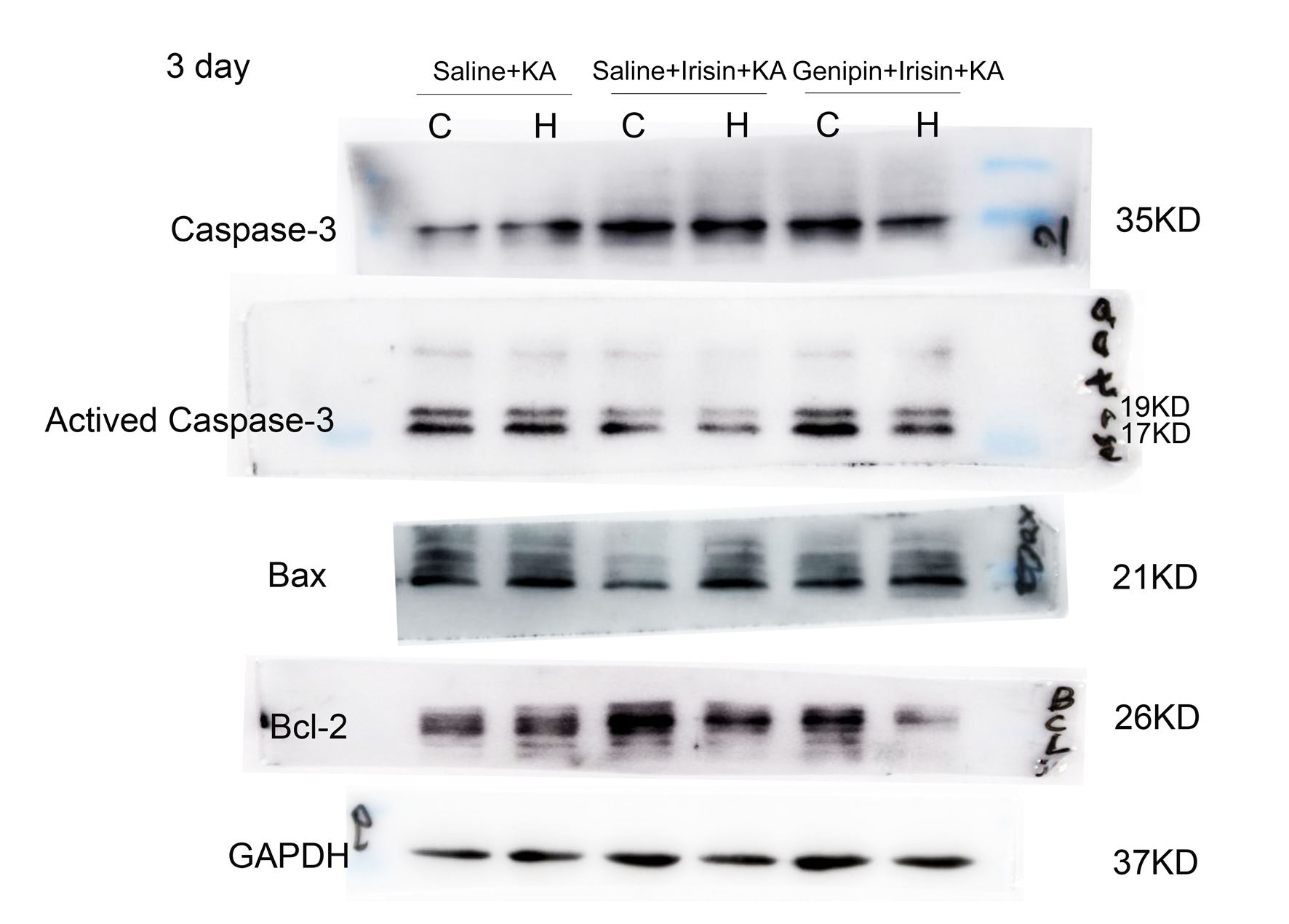

Supplement: Supplementary Figure 5 — The original gel bands of Figure 6. [file Image_5.tif]

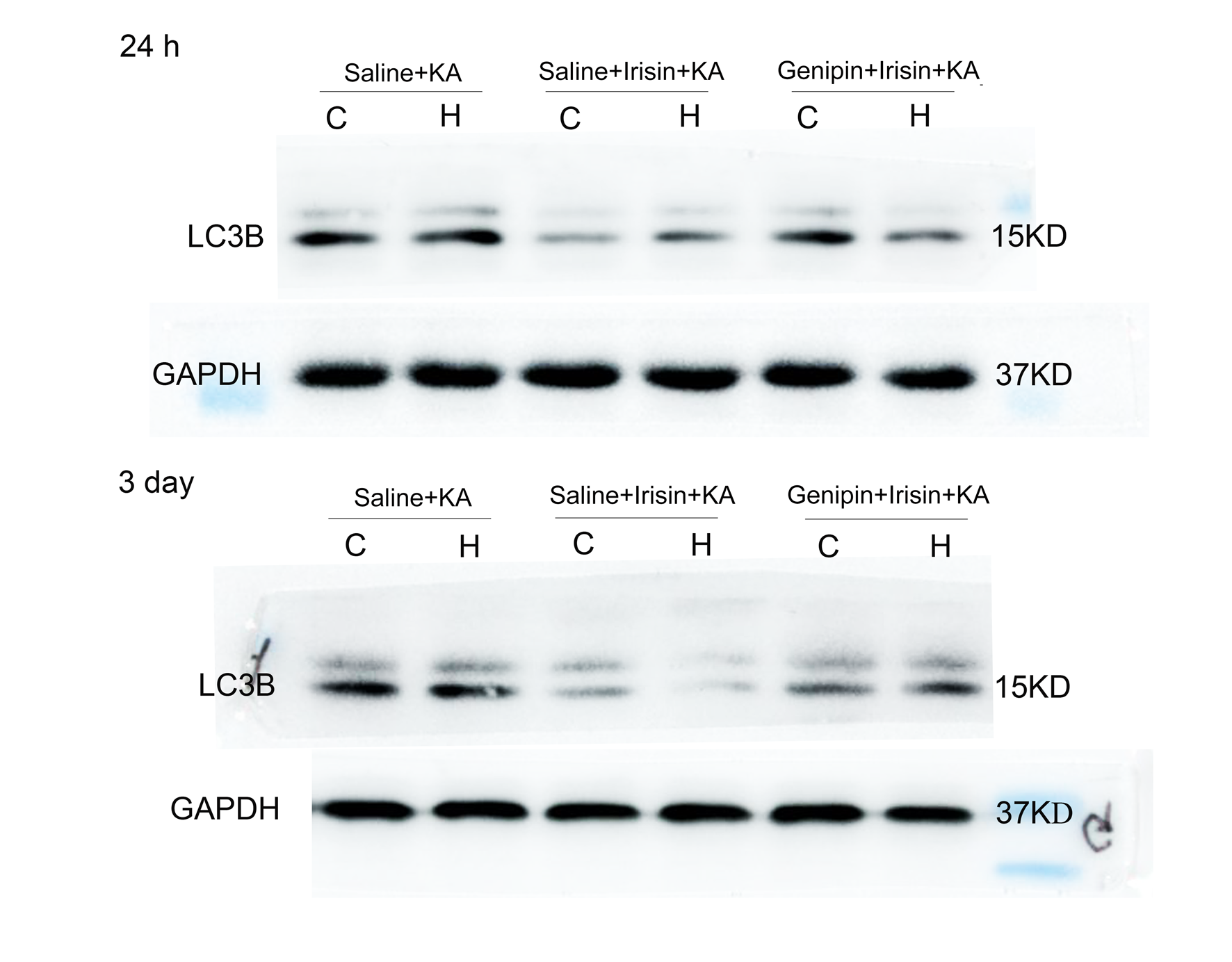

Supplement: Supplementary Figure 6 — The original gel bands of Figure 7. [file Image_6.tif]

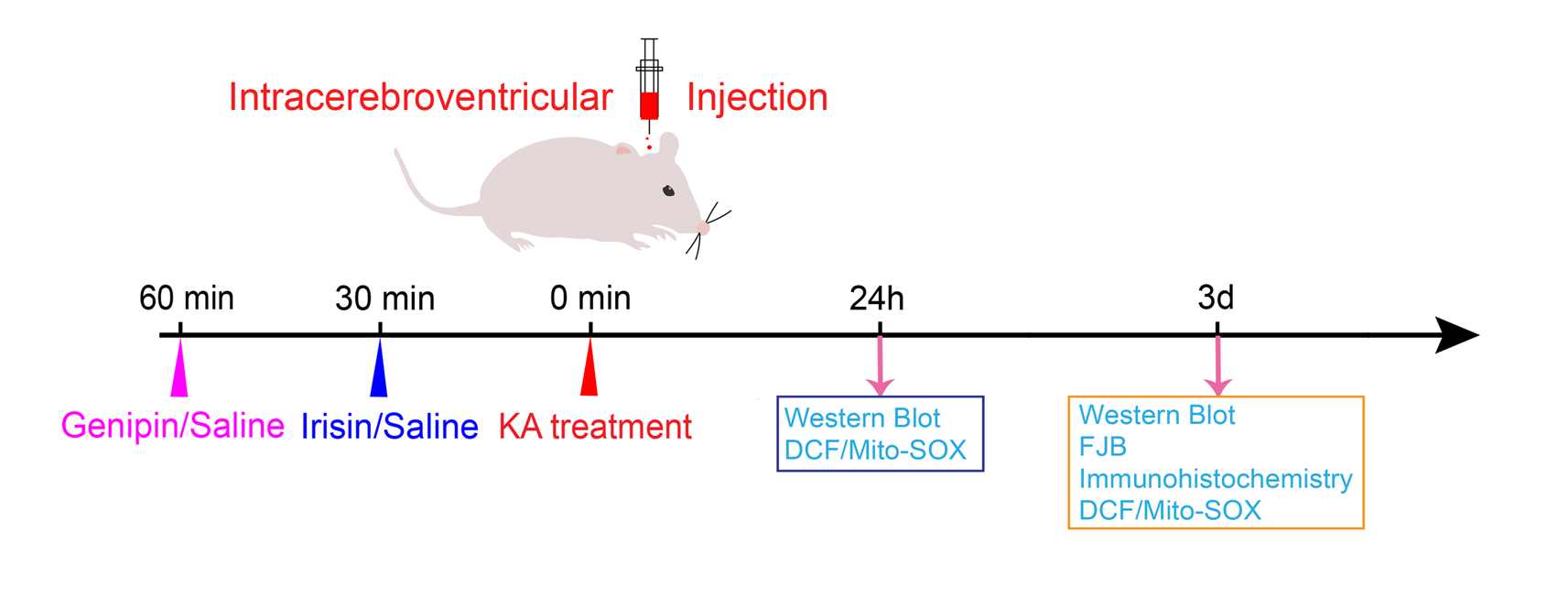

Supplement: Supplementary Figure 7 — Experimental grouping and detailed pharmacological manipulations. [file Image_7.TIF]
